# Studies of *in vivo* speckle contrast imaging based on an improved laser speckle imaging method

**DOI:** 10.1117/1.JBO.30.8.086004

**Published:** 2025-08-26

**Authors:** Guang Han, Qinglong Yang, Rui Zeng, Siyu Liu, Yifan Wu, Ruijuan Chen, Huiquan Wang, Jun Zhang

**Affiliations:** a Tiangong University, School of Life Science, Tianjin, China; b Tianjin Key Laboratory of Control and Evaluation Technology for Medical Devices, Tianjin, China

**Keywords:** blood flow monitoring, fluent imaging technique, small vessel imaging, proximal common carotid artery intervention, ferric chloride (FeCl_3_)-induced artificial embolization

## Abstract

**Significance:**

Laser speckle contrast imaging (LSCI) is widely used for intraoperative blood flow monitoring, but traditional methods have limitations in imaging low blood flow velocities and small vessels. An improved LSCI method, termed the fluent imaging technique, is proposed to enhance imaging sensitivity and accuracy, providing real-time and high-resolution blood flow assessment for neurosurgical applications.

**Aim:**

We aim to validate the performance of the fluent imaging technique in imaging small vessels with low blood flow velocities and assess its application in cerebrovascular surgical procedures, including carotid artery clamping, reperfusion, and ferric chloride ${(\mathrm{FeCl}}_{3})$-induced thrombosis.

**Approach:**

The fluent imaging technique was validated *in vivo* using male Sprague-Dawley rats, with three types of experiments: (1) ear vein vessel imaging, (2) proximal common carotid artery blood flow intervention (stenosis and clamping), and (3) ${\mathrm{FeCl}}_{3}$-induced thrombosis. Blood flow changes were monitored in real time using an LSCI system, and signal-to-background ratio (SBR) analysis was conducted to assess image quality improvements.

**Results:**

The fluent imaging technique improved image quality, particularly for small vessels and low-velocity blood flow, compared with traditional LSCI methods. In capillary regions, it achieved up to 189% improvement in SBR over spatial contrast (SK) and 37% over AWSDK. In a selected region of interest, the SBR increased from 0.53 (SK) and 1.12 (AWSDK) to 1.53 with the fluent imaging method. In carotid artery interventions, the method effectively captured dynamic blood flow changes, including early Relative Blood Flow Index (RBFI) recovery after clamp release. In ${\mathrm{FeCl}}_{3}$-induced thrombosis experiments, it detected vascular occlusion and collateral perfusion.

**Conclusions:**

The fluent imaging technique enhances the accuracy and sensitivity of LSCI for blood flow monitoring in neurosurgery. It provides reliable real-time intraoperative assessment of vascular conditions, improving surgical safety and efficacy. We establish a foundation for its broader clinical application and further optimization.

## 1 Introduction

In neurovascular interventional surgery, vascular visualization technology is crucial for doctors to diagnose and treat vascular diseases and reduce postoperative complications.^1^^,^^2^ Traditional vascular visualization techniques, such as contact Doppler ultrasound and flow velocity measurement, have long been widely used in neurosurgery.

Contact Doppler ultrasound is favored for its cost-effectiveness and ease of application. However, it inherently involves manual operation, which introduces a degree of error, with the accuracy of the results dependent on factors such as the angle of incidence, examination points, and vessel thickness. This makes it a qualitative technique that struggles to provide quantitative data.^2^ In addition, it has limitations in assessing the patency of small penetrating arteries and their impact on neurological outcomes. Furthermore, clinical experience indicates that Doppler sensors are prone to damage and are not suitable for frequent disinfection.^3^ The ultrasonic flowmeter has demonstrated exceptional performance in evaluating anastomotic blood flow and detecting large vessel occlusion in cases of aneurysmal dissection due to its ability to measure volumetric blood flow velocity.^2^ This is particularly crucial for patients with large and giant aneurysms. Indocyanine green angiography (ICG) has garnered widespread attention in vascular neurosurgery for its simplicity and noninvasive nature.^4^^–^^6^ However, this technique also has limitations, primarily in that it can only track fluorescent blood flow within the visible range. In addition, obstacles such as vascular branches, clamps, or aneurysms can restrict its effectiveness. Modern multimodal operating rooms, such as VISIUS and AMIGO, integrate magnetic resonance angiography (MRA) and computed tomography angiography (CTA). Although these technologies provide comprehensive vascular imaging, their preparation and examination times pose logistical challenges in the operating room, limiting the simultaneous execution of imaging and interventions during surgery.

Laser Speckle contrast imaging (LSCI) is a promising method for evaluating blood flow during neurosurgical procedures.^7^ LSCI measures blood flow by calculating the speckle contrast value, which is quantitatively related to the velocity of scattering particles within a given field.^8^^–^^12^ This allows us to determine the relative blood flow speed at each pixel within the field, creating a color-coded image where the intensity represents the relative blood flow speed. However, traditional LSCI imaging methods, such as the time contrast analysis method (TK) and the spatial contrast (SK) analysis method, are not ideal for imaging low blood flow or small vessels.^13^^,^^14^ The low flow speeds result in insignificant changes in the speckle pattern, leading to suboptimal spatial contrast analysis and the inability to accurately image small vessels and measure blood flow velocity. To enhance the imaging depth of LSCI, our research team proposed an adaptive window spatial direction contrast (AWSDK) method.^15^^,^^16^ This approach improves the imaging quality of deep vessels while maintaining temporal resolution. To address the susceptibility of traditional methods to static scattering, our research team proposed an improved laser speckle contrast imaging method,^17^ referred to as the fluent imaging technique. This technique aims to minimize the impact of static scattering and has been validated for its effectiveness through experiments involving phantoms, mice, and rabbit surface models. The aim of this study is to verify the significant improvement of the fluent imaging technology in imaging low-velocity small blood vessels and to evaluate the effectiveness of this improved LSCI method in common carotid artery procedures during neurosurgery, such as proximal stenosis, clamping, reperfusion, and intraoperative vascular thrombosis formation. By validating the blood flow assessment capabilities of LSCI under these standard cerebrovascular surgical conditions, the aim is to provide more reliable and real-time technical support for intraoperative blood flow monitoring, thereby enhancing the safety and efficacy of surgeries.

## 2 Materials and Methods

### 2.1 Animal Preparation

Before conducting the animal experiments, approval was obtained from the Ethics Committee of the Institute of Radiation Medicine, Chinese Academy of Medical Sciences, and the study adhered to the guidelines for humane animal care.

All experiments were conducted using 2-month-old male Sprague-Dawley (SD) rats weighing 330 to 350 g (n=9). Each experimental group consisted of three rats, assigned to the following experiments: ear vein vascular assessment (experiment 1), proximal common carotid artery (CCA) blood flow intervention (experiment 2), and ferric chloride (${\mathrm{FeCl}}_{3}$)-induced artificial embolism (experiment 3). Prior to the experiments, rats were randomly grouped based on body weight by a veterinarian (the weight difference did not exceed 10%).

The rats were isolated and housed under standard conditions for 2 weeks to ensure their health status. After the isolation period, the veterinarian conducted health assessments following Good Laboratory Practice (GLP) principles, excluding any animals showing signs of disease. No animals were excluded after the isolation period.

During the experiments, rats were anesthetized using pentobarbital sodium (Sigma-Aldrich, USA) at a dose of 50 mg/kg, administered via intraperitoneal injection (IP). If the depth of anesthesia was insufficient (as indicated by a response to pain stimuli), an additional dose of pentobarbital sodium (20 mg/kg, IP) was administered.

For experiment 1, after anesthesia, the rats were placed in the supine position, and their heads and limbs were secured using a fixation device to prevent movement during imaging. The hair on the marginal ear vein was shaved, and the surface was cleaned with sterile gauze or cotton balls to remove excess oil and dirt.

For experiments 2 and 3, all rats were similarly placed in the supine position and secured on the surgical table. The surgical area on the neck was disinfected, and the hair was shaved to ensure a sterile environment. Under a microscope, a longitudinal incision was made to expose the bilateral CCAs by carefully separating the connective tissue around the vessels using micro forceps and scissors, ensuring the vessels were fully exposed without damage.

In experiment 2, a microvascular clamp was used to occlude the right CCA, and blood flow changes were recorded before and after the occlusion. After 60 s of occlusion, the clamp was removed, and the recovery of blood flow was observed and recorded. Subsequently, the proximal CCA was clamped, and blood flow changes were recorded before and after the clamping. The clamp was removed after 40 s, and the recovery of blood flow was observed and recorded.

In experiment 3, a piece of filter paper soaked in 30% ${\mathrm{FeCl}}_{3}$ solution was gently placed on the surface of the exposed right CCA for ∼5 min. The filter paper was then removed, and the area was rinsed with saline. Blood flow changes were observed and recorded. Both experiments were performed on the carotid arteries of the rats, which are approximately the same size as the cerebral arteries (1  mm±0.1  mm). The bilateral CCAs were extensively isolated in the rats, and a latex thin liner was placed underneath the vessels for better observation.

To conduct hypothesis testing on the samples, the Relative Blood Flow Index (RBFI, unit: a.u.) was used as the primary evaluation metric. The RBFI is calculated as $\mathrm{RBFI}=\frac{1}{\tau_{c}}$, where $\tau_{c}$ is the speckle correlation time, representing the average time over which the speckle pattern remains correlated and is inversely related to the particle velocity. To verify the imaging quality, the signal-to-background ratio (SBR) was introduced, and its calculation formula is as follows:

$$\mathrm{SBR}=\frac{\left|K_{\mathrm{signal}}-K_{\mathrm{background}}\right|}{K_{\mathrm{background}}},$$ (1)

where $K_{\mathrm{signal}}$ is the speckle contrast within the region of interest (vessel area) and $K_{\mathrm{background}}$ is the speckle contrast of the background (nonvessel tissue). In the improved LSCI method, the RBFI was first computed from the inverse correlation time $\frac{1}{\tau_{c}}$, which was estimated from the speckle contrast using the established analytical model. For quantitative comparison, the SBR was then calculated based on the RBFI maps rather than directly from the speckle contrast, in order to evaluate the final flow-resolved images.

### 2.2 Experimental System and LSCI Execution

**Fig. 1:**
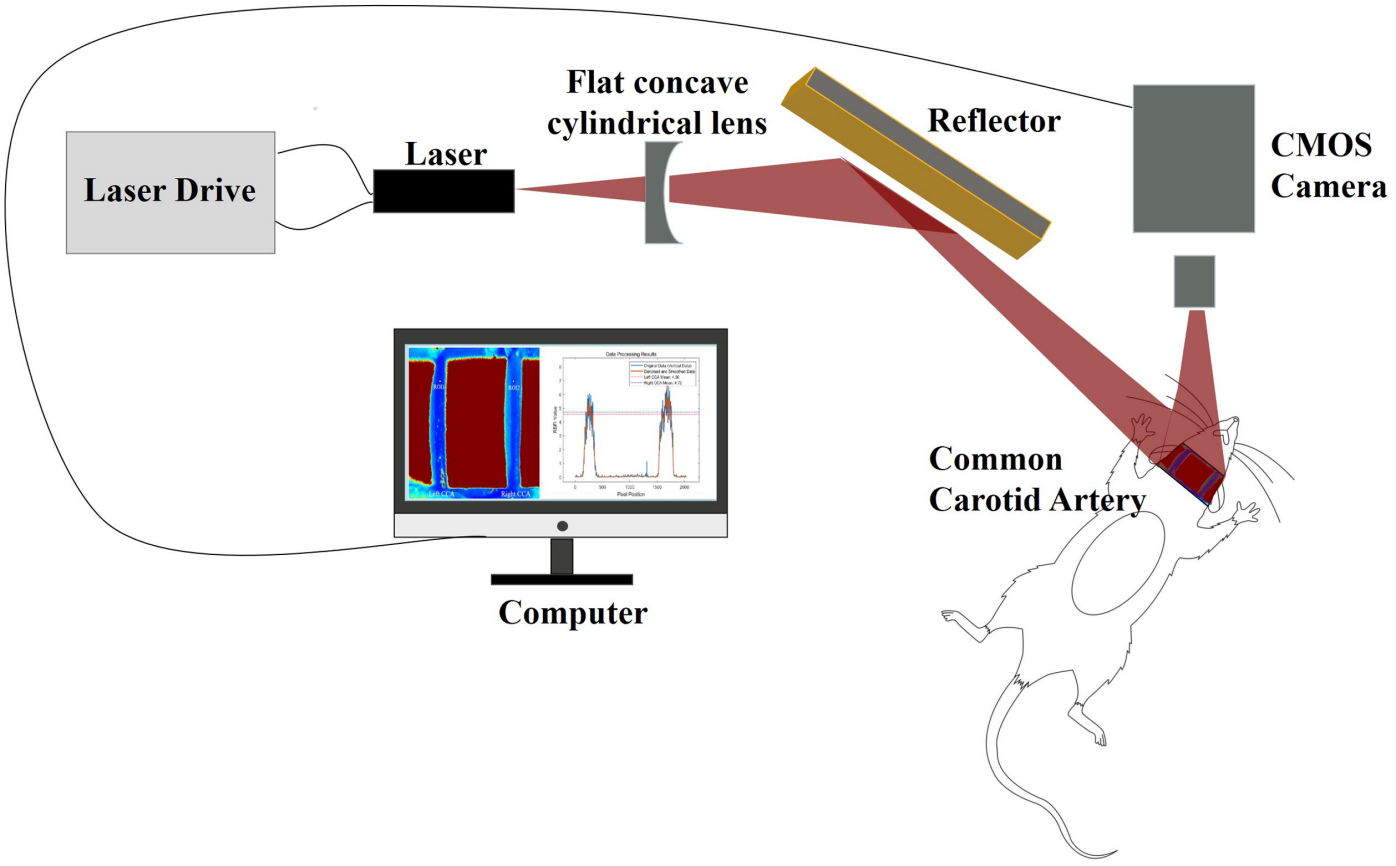
Diagram of the experimental setup.

The experimental system used a self-developed LSCI imaging system, which included a Zyla 4.2 sCMOS camera (Andor, UK), a CFI Super Fluor objective lens (4×/0.20, WD = 15.5 mm) (Nikon, Japan), and a 785-nm laser diode (PH785DBR180T8, Photodigm, USA), with output power controlled by a laser driver (LDC-3908, ILX Lightwave, USA). The camera’s exposure time was set to 5 ms in all experiments. The laser radiation was scattered and expanded through a series of optical components, including a convex lens (LB1630-B, Thorlabs, USA), a plano-concave cylindrical lens (GL16, Golden Way Scientific, China), and mirrors (ME2S-M01, Thorlabs, USA), before being directed onto the experimental subject. The scattered coherent light was projected onto the CMOS camera through the objective lens, and a computer processed the acquired speckle images to generate speckle contrast images. The experimental setup used for both experiments 1 and 2 is illustrated in Fig. 1. As shown, this configuration corresponds to experiment 2, in which blood flow imaging was performed on the exposed carotid artery.

In LSCI, blood flow is measured using the contrast ratio, which is the ratio of the standard deviation to the mean of the speckle light intensity. After obtaining the raw speckle images, spatial or temporal contrast can be computed based on the type of window selected for calculation. The speckle contrast is a function of the exposure time *T* ^18^ and is related to the correlation time of the speckle light intensity correlation function, as expressed by the following equation:

$$K^{2}=\beta \{\frac{\tau_{c}}{T}+\frac{\tau_{c}^{2}}{2T^{2}}[(\sqrt{\frac{-2T}{\tau_{c}}})-1]\}.$$ (2)

In Eq. 2, $\tau_{c}$ represents the correlation time of the speckle intensity function and β is a constant factor determined by the imaging system that is related to the ratio of detector size to speckle size and polarization effects.^19^

The contrast of speckles can be used to study the motion of blood cells for several physical reasons. First, the observed speckle blurring includes both dynamic speckle patterns caused by moving blood cells and static speckle components arising from stationary tissue, which may interfere with accurate flow assessment. Second, the reciprocal of the speckle correlation time $\frac{1}{\tau_{c}}$ is proportional to the velocity of scattering particles, providing a quantitative link between contrast and flow. Finally, when the exposure time T is sufficiently long compared with $\tau_{c}$, Eq. (2) can be approximated as $\frac{1}{\tau_{c}}\propto \frac{1}{K^{2}}$.^20^^,^^21^ This relationship allows direct estimation of relative blood flow index (RBFI) from the speckle contrast K, simplifying the analysis in practical applications.

Throughout the entire experiment, the exposure time for each frame of the speckle image was set to 5 ms, enabling a frame rate of 100 fps in full-frame mode. When region-of-interest (ROI) acquisition was used, the frame rate could be increased up to 200 fps. For the speckle images at each exposure time, the spatial speckle contrast of each frame was first calculated using a 7 × 7 sliding window. Then, the average spatial speckle contrast over 30 consecutive frames was computed. The temporal speckle contrast value for the 30 frames was obtained using the temporal contrast analysis algorithm.

The relationships between $K_{s}$ and $K_{t}$^22^^,^^23^ with the autocorrelation time are as follows:

$$K_{s}^{2}=[\beta \rho^{2}\frac{e^{-2x}-1+2x}{2x^{2}}+4\beta \rho (1-\rho )\frac{e^{-x}-1+x}{x^{2}}+\beta (1-\rho {)}^{2}]+V_{\mathrm{noise}},$$ (3)

$$K_{t}^{2}=[\beta \rho^{2}\frac{e^{-2x}-1+2x}{2x^{2}}+4\beta \rho (1-\rho ){\frac{e^{-x}-1+x}{x^{2}}}^{2}]+V_{\mathrm{noise}}.$$ (4)

$K_{s}$ and $K_{t}$ represent the spatial and temporal speckle contrast values, respectively. The parameter ρ denotes the proportion of dynamically scattered light to the total scattered light, representing the dynamic scattering ratio. $x=\frac{T}{\tau_{c}}$ represents the ratio of the exposure time T to the speckle correlation time $\tau_{c}$. $V_{\mathrm{noise}}$ accounts for the variance introduced by the imaging system noise.

Due to the different impacts of static scattering on spatial contrast analysis and temporal contrast analysis, the proportion of dynamically scattered light to the total scattered light, denoted as ρ, can be obtained by combining Eqs. (3) and (4). The expression is given by

$$\rho =1-\sqrt{\frac{K_{s}^{2}-K_{t}^{2}}{\beta }}.$$ (5)

By substituting ρ into $K_{s}$, we can derive the correlation time function $\tau_{c}$.

This method allows us to effectively eliminate the impact of static scattering on single-exposure laser speckle contrast imaging, thereby improving the accuracy and reliability of blood flow monitoring. The approach simplifies the data acquisition and processing workflow, eliminating the need to collect speckle images at multiple exposure times for nonlinear fitting. This significantly shortens the time required to monitor blood flow changes, making the monitoring process faster and more practical.

## 3 Result

### 3.1 Microvascular Imaging Evaluation

**Fig. 2:**
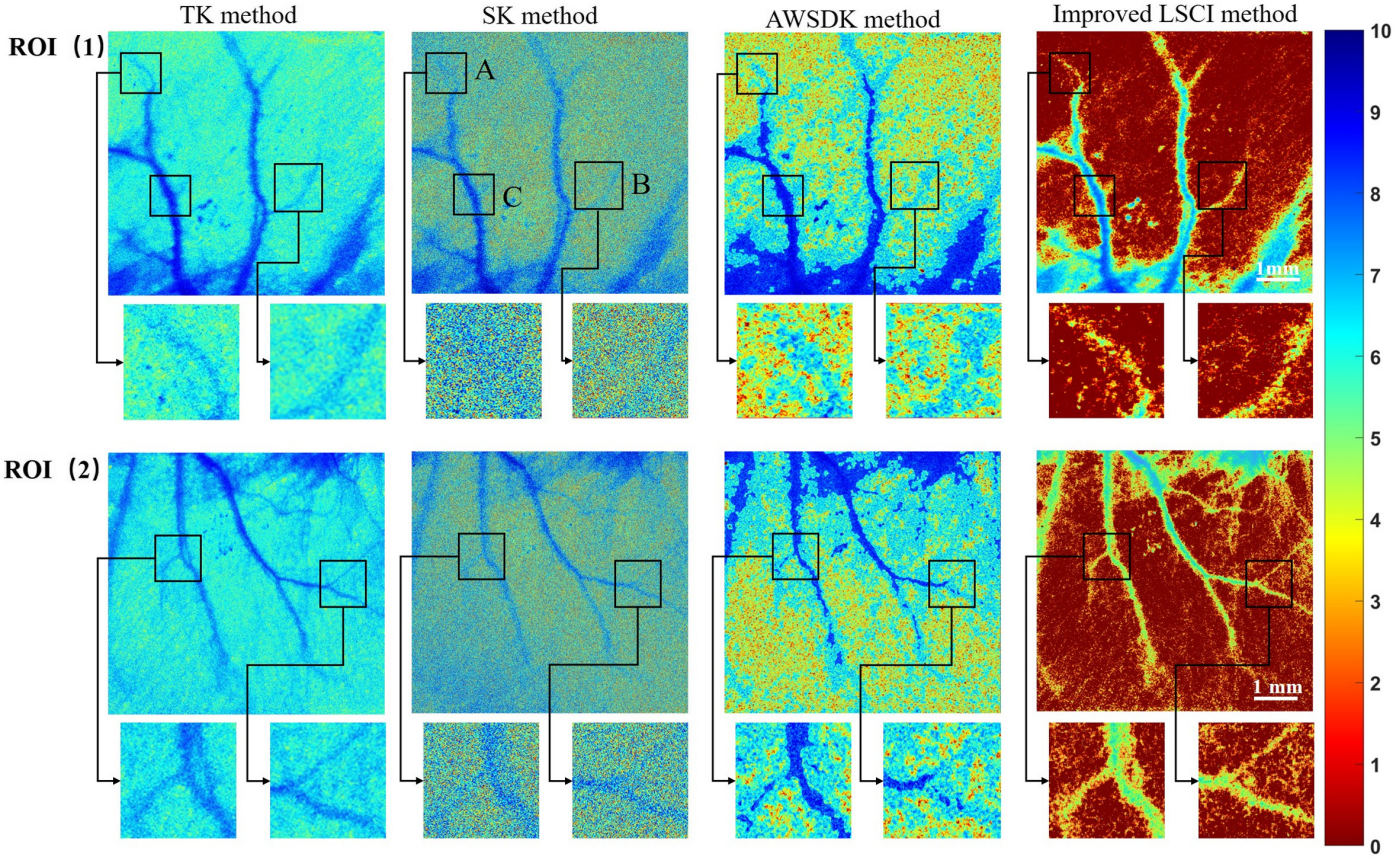
Comparison of blood vessel visualization in the ears of two SD rats using the temporal contrast (TK), spatial contrast (SK) method, adaptive window spatial direction contrast (AWSDK), and the fluent imaging technique (n=2  rats).

In this experiment, a reflective LSCI system was used to capture images of two different regions on the rat ear, labeled as ROIs (1) and (2). Traditional LSCI methods often yield blurred results when imaging the rat ear area, making it difficult to distinguish between blood vessels and other tissues clearly. However, with our improved LSCI method, significantly enhanced pseudo color images were obtained, allowing for clear differentiation between blood vessels and other tissues, and showcasing more detailed features of the blood vessels. As shown in Fig. 2, a systematic evaluation of the fluent imaging technique proposed in this study was performed through comparative experiments with the traditional spatial contrast (SK) method, the adaptive window spatial direction contrast (AWSDK) method, and the fluent imaging method.

**Fig. 3:**
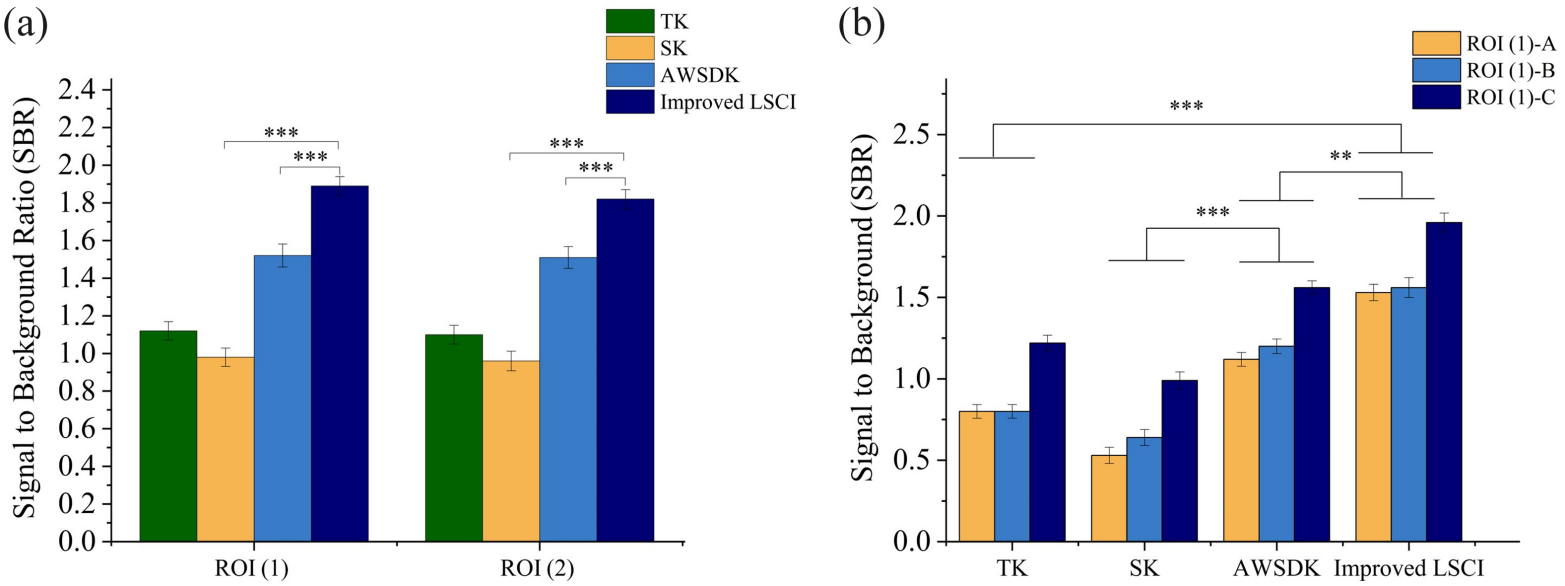
Quantitative comparison of the SBR among different imaging methods: (a) comparison of SBR between ROI (1) and ROI (2), and (b) SBR analysis of three vascular regions (A, B, C) selected within ROI (1). Error bars indicate the standard deviation. Statistical significance: **p<0.01 and ***p<0.001.

To further compare the performance of traditional imaging methods and the fluent imaging technique, the signal-to-background ratio (SBR) of ROIs (1) and (2) was analyzed. As shown in Fig. 3(a), in ROI (1), the fluent imaging method achieved an SBR of 1.89±0.05, representing an improvement of 69% compared with the TK method, 93% compared with the SK method, and 24% compared with the AWSDK method. In ROI (2), the SBR obtained with the fluent imaging method was 1.82±0.05, corresponding to an improvement of 66% over the TK method, 90% over the SK method, and 21% over the AWSDK method (p<0.001). To further assess the enhancement of vascular details, three vessel regions (A, B, and C) within ROI (1) were selected, as shown in Fig. 3(b). SBR analysis of these regions demonstrated that the fluent imaging technique outperformed both the SK and AWSDK methods in terms of vessel contrast enhancement and background suppression. Specifically, in region A, the fluent imaging method improved the SBR by 91% compared with the TK method, by 189% compared with the SK method, and by 37% compared with the AWSDK method; in region B, the improvements were 95%, 144%, and 30%, respectively; and in region C, the improvements were 61%, 98%, and 26%, respectively (p<0.01 for all comparisons). These results fully validate that the fluent imaging technique significantly enhances overall imaging contrast and the visualization of microvascular details compared to existing LSCI methods.

### 3.2 Blood Flow Assessment for Right CCA Stenosis and Clamping Experiment

In this experiment, the improved LSCI method (fluent imaging technique) was used to validate the changes in distal blood flow of the right CCA under the background of proximal CCA blood flow intervention. The experimental design included two types of interventions: stenosis and clamping, with the aim of evaluating the dynamic changes in blood flow and perfusion response.

**Fig. 4:**
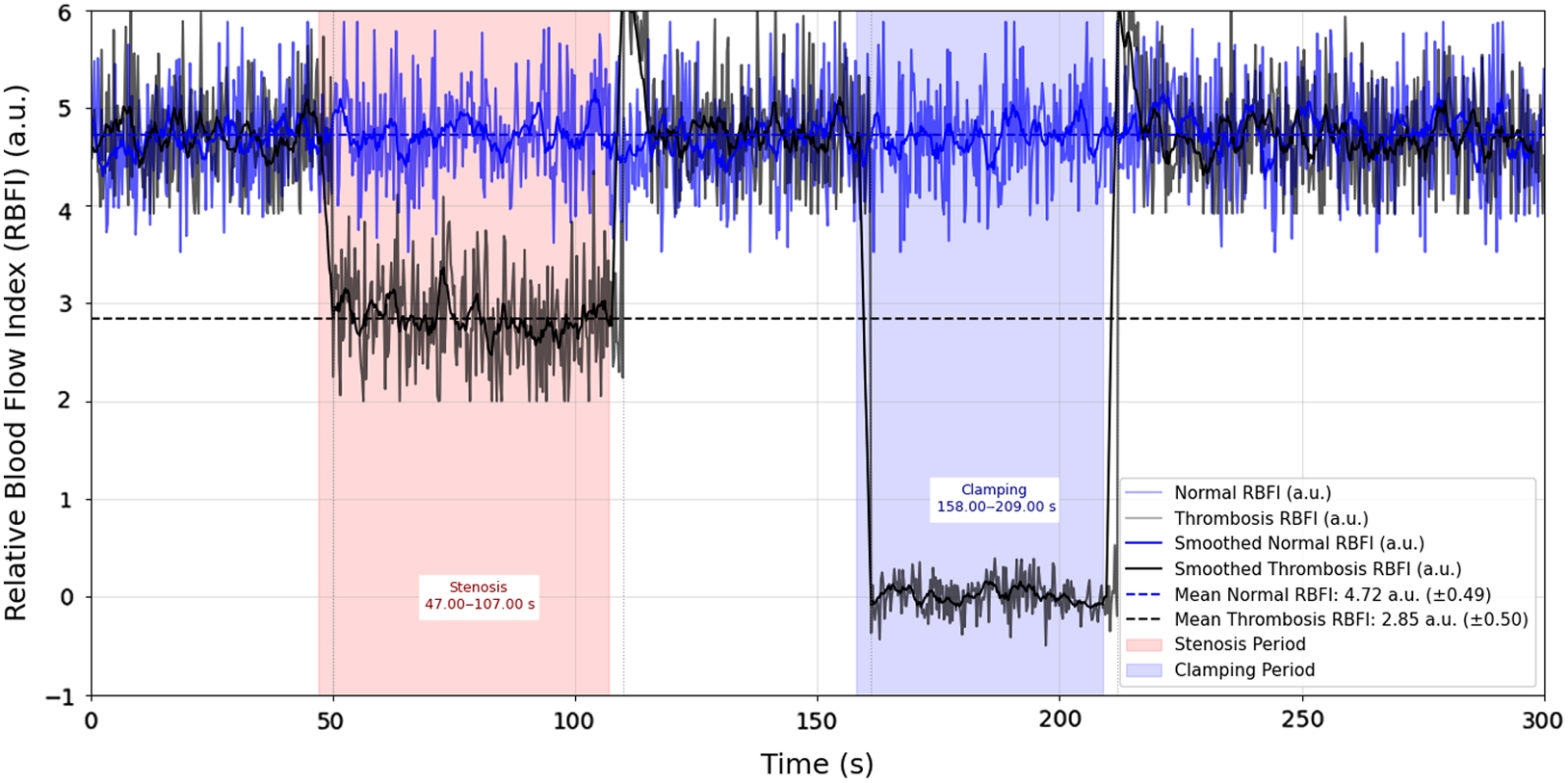
Temporal changes in RBFI under normal and thrombosis conditions in SD rats (n=3  rats).

As shown in Fig. 4, this study meticulously recorded and analyzed the RBFI changes in the right CCA throughout the experiment. The figure marks the normal RBFI variation during the first 300 s before the experiment and the changes in RBFI during the experimental process. The baseline data for the first 300 s demonstrate the fluctuations in RBFI in the rat under stable conditions. It can be observed that the RBFI remained relatively stable during the baseline period. During the experiment, significant changes in the RBFI of the right carotid artery occurred. The experimental phases included stenosis and clamping. During these interventions, the RBFI decreased significantly and exhibited different trends at various stages of the experiment.

**Fig. 5:**
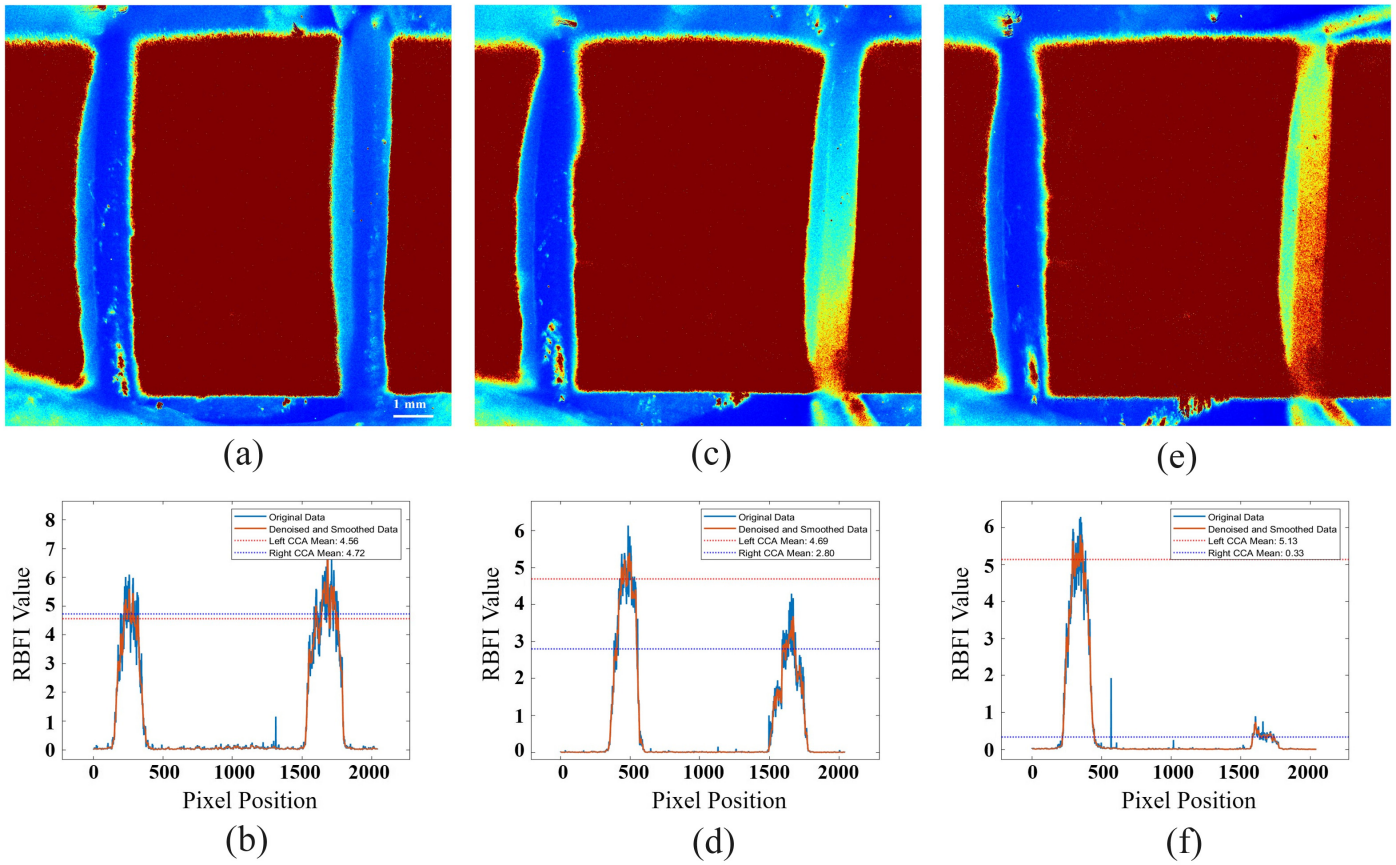
Continuous LSCI monitoring of bilateral common carotid arteries (CCAs) during different interventions in SD rats (n=3  rats): (a) LSCI image of normal bilateral CCAs, (b) RBFI of normal bilateral CCAs, (c) LSCI image of right proximal CCA stenosis experiment, (d) RBFI of right proximal CCA stenosis experiment, (e) LSCI image of right proximal CCA clamping experiment, and (f) RBFI of right proximal CCA clamping experiment.

In the stenosis experiment, the average RBFI of the right common carotid artery (CCA) was first measured before the stenosis, as shown in Figs. 5(a) and 5(b). The initial RBFI was 4.72 a.u. (±0.49). After applying the stenosis to the right CCA, the RBFI significantly decreased to 2.84 a.u. (±0.50), a reduction of ∼40%, as shown in Figs. 5(c) and 5(d). At 107 s, when the stenosis was removed, the RBFI suddenly surged, exceeding the normal RBFI, and then gradually returned to the baseline level. This sudden increase in perfusion indicates enhanced vascular reactivity, caused by the rapid influx of blood following the removal of the stenosis.

In the clamping experiment, RBFI changes in the distal right CCA were evaluated under the background of proximal clamping. After the proximal clamping, the distal RBFI rapidly decreased to 0.33 a.u. (±0.12), as shown in Figs. 5(e) and 5(f). Furthermore, we were able to identify the presence and intensity of retrograde blood flow under the clamping condition and detect regions with no blood flow, distinguishing between weak retrograde flow and areas with no flow. After the clamp was removed, blood flow returned to previous levels, with a slight increase in perfusion, and the RBFI recovered to 4.72 a.u. (±0.49). This recovery phenomenon, similar to the blockage experiment, indicates significant vascular reactivity following clamping.^24^^–^^26^

In the stenosis and clamping experiments, the RBFI in the left CCA increased from 4.56 a.u. (±0.45) to 4.69 a.u. (±0.46) and 5.13 a.u. (±0.50), respectively. When the right CCA was subjected to intervention, the left CCA compensated for the reduction in right-sided blood flow by increasing blood supply through collateral circulation, fulfilling the compensatory effect of collateral blood flow.

In this study, a t-test was used to evaluate the differences in RBFI between the left and right CCAs under normal conditions. The t-test is suitable for comparing the mean differences between two independent samples, particularly when the sample size is small and the data are approximately normally distributed. Before conducting the t-test, the normality of the data was confirmed using the Shapiro-Wilk test (p>0.05), ensuring that the data met the prerequisites for the t-test.

The results showed that the average RBFI of the left and right CCAs was 4.56 a.u. (±0.45) and 4.72 a.u. (±0.49), respectively, with a difference of 0.16 a.u. The t-test results (t=−0.828, p=0.421) indicated that this difference was not statistically significant (p>0.05), suggesting that under normal conditions, the RBFI difference between the left and right CCA can be considered negligible, falling within the range of normal physiological variation. Specific results are shown in Table 1.

**Table 1:** Blood flow variations and statistical analysis for right CCA interventions.

Condition pair	Mean RBFI (a.u.)	Standard deviation	Standard error	Test method	t-value/*p*-value
Left CCA, normal	4.56	0.45	0.142	T-test	t=−0.828, p=0.421
Right CCA, normal	4.72	0.49	0.155		
Right CCA, pre-stenosis	4.72	0.49	0.155	T-test	t=19.49, p<0.001
Right CCA, post-stenosis	2.84	0.50	0.158		
Right CCA, pre-clamping	4.72	0.49	0.155	T-test	t=26.88, p<0.001
Right CCA, post-clamping	0.33	0.12	0.038		
Left CCA, pre-stenosis	4.56	0.45	0.142	Mann–Whitney U test	p<0.05
Left CCA, post-stenosis	4.69	0.46	0.145		
Left CCA, pre-clamping	4.56	0.45	0.142	Mann–Whitney U test	p<0.05
Left CCA, post-clamping	5.13	0.50	0.158		

Through this set of experiments, it can be observed that the fluent imaging technique has high sensitivity in detecting changes in blood flow. Whether in the case of blockage or clamping, it accurately detects significant decreases in blood flow and the perfusion response after the intervention is removed. Using this method, can be achieve real-time monitoring of dynamic blood flow changes.

### 3.3 Blood Flow Assessment for Artificial Embolization Experiment

**Fig. 6:**
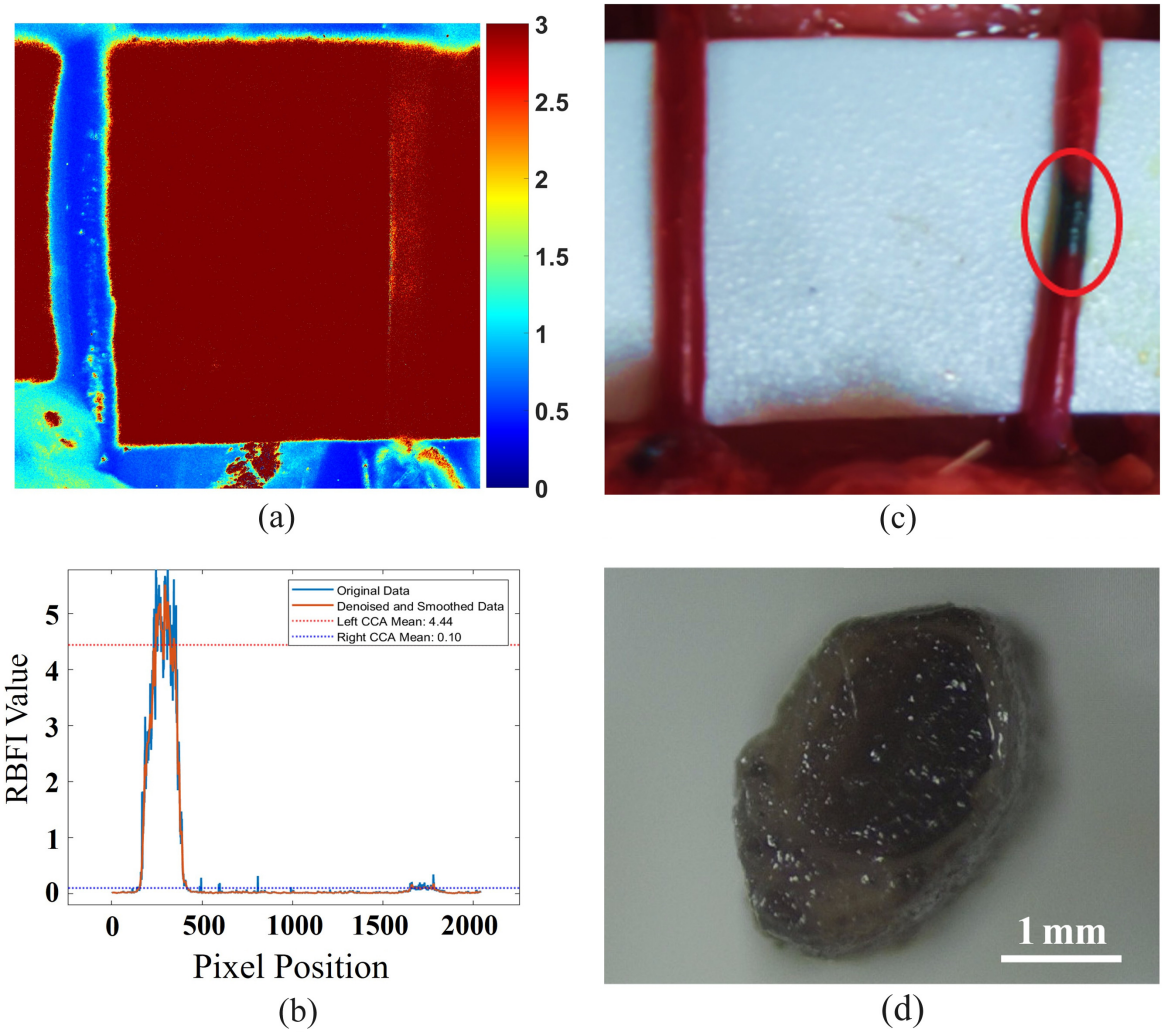
Images from the artificial embolization experiment (n=3  rats): (a) LSCI image of the artificial embolization experiment, (b) RBFI of the artificial embolization experiment, (c) visible light image of the artificial embolization experiment, and (d) cross-sectional view of the embolized vessel.

In this study, blood flow changes in both the right and left CCAs were assessed under normal conditions and after ${\mathrm{FeCl}}_{3}$-induced thrombosis. The experimental results are shown in Fig. 6 and Table 2.

**Table 2:** Blood flow changes and statistical analysis in normal state and ${\mathrm{FeCl}}_{3}$-induced thrombosis experiment.

Condition pair	Mean RBFI (a.u.)	Standard deviation	Standard error	Test method	t-value/p-value
Right CCA, normal	4.86	0.25	0.079	T-test	t=−0.499, p=0.625
Left CCA, normal	4.78	0.30	0.095		
Right CCA, pre-thrombosis	4.86	0.25	0.079	T-test	t=23.45, p<0.001
Right CCA, post-thrombosis	0.10	0.08	0.025		
Left CCA, pre-thrombosis	4.78	0.30	0.095	T-test	t=−4.89, p<0.05
Left CCA, post-thrombosis	5.17	0.35	0.111		

In the normal state, the mean blood flow in the right CCA was 4.86 a.u. (±0.25) and the mean blood flow in the left CCA was 4.78 a.u. (±0.30), with a minimal difference of 0.08 a.u. (t=−0.499, p=0.625), which was not statistically significant. This indicates that the blood flow in both arteries is quite similar under normal conditions.

Following the ${\mathrm{FeCl}}_{3}$-induced thrombosis, the right CCA blood flow decreased significantly from 4.86 a.u. (±0.25) to 0.10 a.u. (±0.08), indicating almost complete occlusion (t=23.45, p<0.001). Meanwhile, the left CCA demonstrated a compensatory collateral circulation effect, with blood flow increasing from 4.78 a.u. (±0.30) to 5.17 a.u. (±0.35), showing a significant compensation for the reduced blood flow in the right CCA (t=−4.89, p<0.05).

Through this study, we validated the effectiveness and reliability of the fluent imaging technique in detecting and assessing intravascular thrombosis, providing strong support for real-time monitoring and evaluation of blood flow changes during surgery.

## 4 Discussion

The results of the study indicate that the improved LSCI method allows for real-time assessment of vascular blood flow interference under standard conditions commonly encountered in cerebrovascular surgeries, such as temporary arterial clamping, arterial clamping, microvascular anastomosis patency, and intraoperative arterial thrombosis. Although motion artifacts occurred during surgical manipulation, the imaging system maintained sufficient stability to support reliable observation of blood flow changes. This was facilitated by the short exposure time (5 ms), high frame rate (100 to 200 fps) acquisition, and speckle averaging, which together improved robustness against minor motion. The observed motion during imaging was limited to less than 1.5 mm, mainly due to minor physiological movements under anesthesia. In typical clinical applications, expected motion due to respiration or cardiac activity may reach 1 to 3 mm. Therefore, our model represents a relatively mild but realistic motion scenario for validating robustness against motion artifacts.

In this study, we define small vessels as those with diameters less than 100  μm, including capillaries and small venules. Low flow regions refer to areas with relative blood flow velocities in the range of 0.2 to 1 mm/s. By quantitatively comparing the traditional spatial contrast (SK) method, the adaptive window spatial direction contrast (AWSDK) method, and the improved LSCI method, we observed a significant improvement in imaging performance, particularly for small blood vessels with low blood flow. As shown in Fig. 2, the fluent imaging technique provided clearer vessel boundaries and enhanced microvascular detail. This was supported by quantitative SBR analysis in Fig. 3. These results demonstrate that the fluent imaging technique is particularly effective for visualizing small, low-flow vessels, outperforming existing LSCI methods under challenging scattering conditions in biological tissues.

Experimental observations indicate that the fluent imaging technique effectively captures dynamic changes in blood flow under both stenosis and clamping conditions. The method successfully visualized the significant reduction in perfusion during occlusion and the recovery of flow following the release of the intervention. In the stenosis experiment, RBFI significantly decreased after the blockage and rapidly recovered upon removal, indicating increased vascular reactivity due to the rapid influx of blood post-blockage. In the clamping experiment, RBFI was observed to rapidly decrease to 0.33 a.u. (±0.12) after proximal clamping and return to initial levels upon removal of the clamp, demonstrating significant vascular reactivity.

In the ${\mathrm{FeCl}}_{3}$-induced thrombosis experiment, the RBFI in the distal left common carotid artery (CCA) decreased to 0.1 a.u. (±0.08) after thrombosis formation, indicating complete intraluminal occlusion. These results demonstrate that the LSCI method can effectively detect changes in blood flow in the left CCA using the FeCl_3_-induced thrombosis model, especially in cases of complete interruption of blood flow.

## 5 Conclusion

This study validates the efficacy of the improved LSCI method (fluent imaging technique), which addresses the limitations of traditional LSCI methods, particularly in imaging low-velocity and small vessels. The experiments on male SD rats under various cerebrovascular conditions—such as proximal stenosis, clamping, reperfusion, and intraoperative thrombosis—showed that the fluent imaging technique significantly enhances image quality and provides higher sensitivity and accuracy in detecting blood flow changes. This study lays a solid foundation for the widespread clinical application of LSCI and offers important references for future research and technological improvements.

## Data Availability

The data that support the findings of this article are not publicly available due to privacy. They can be requested from the author at zhangjun@tiangong.edu.cn.
